# Gender, age, and socioeconomic position-specific associations between illness perception and diabetes-related distress: cross-sectional analysis among people with type 2 diabetes

**DOI:** 10.3389/fendo.2026.1898218

**Published:** 2026-08-31

**Authors:** Elvire N. Landstra, Fleur de Klerk, Dinah van Schalkwijk, Henry Jansen, Lars. L.F.G. Valke, Tom Smeets, Ruth E. Mark, Sabita S. Soedamah-Muthu

**Affiliations:** 1 Department of Medical and Clinical Psychology, Center of Research on Psychological Disorders and Somatic Diseases (CoRPS), Tilburg University, Tilburg, Netherlands; 2 Department of Internal Medicine, Jeroen Bosch Hospital, Den Bosch, Netherlands; 3 Department of Internal Medicine, Elisabeth TweeSteden Hospital, Tilburg, Netherlands; 4 Department of Cognitive Neuropsychology, Tilburg University, Tilburg, Netherlands; 5 Institute for Food, Nutrition and Health, University of Reading, Reading, United Kingdom

**Keywords:** age factors, attitudes, cross-sectional analysis studies, type 2 diabetes mellitus, sex factors, socioeconomic factors, stress, psychological

## Abstract

**Aim:**

Around one-third of people with type 2 diabetes (T2DM) suffer from diabetes-related distress (DRD). Understanding risk factors could aid in targeted interventions to lower distress and improve quality of life. Although the way people perceive their illness – illness perceptions – have been related to DRD, studies so far have been small and it is unclear whether gender, age and socioeconomic position (SEP) affect the association. Therefore, this study aims to investigate the age-, gender-, and SEP-specific relationship between illness perceptions and DRD.

**Methods:**

Analyses were based on cross-sectional data from people with T2DM (n= 1,215, 53.8% men, mean age 63.4). Illness perceptions were measured using the brief Illness Perception Questionnaire (B-IPQ) and DRD with the Problem Areas In Diabetes (PAID-5). We related illness perception scores and perceived causes of T2DM to DRD using linear regression models, adjusting for gender, age, SEP, and diabetes duration. Interactions with age, gender, and SEP were tested, and stratified analyses were run if significant.

**Results:**

One SD increase in the illness perception score related to a 2.89 (95% confidence interval (CI): 2.72; 3.06) increase in DRD. Women and people below 60 years had a stronger relation between negative illness perceptions and DRD compared to men and older people. People attributing T2DM to psychological causes compared to non-modifiable ones had more DRD.

**Conclusions:**

Overall, a more threatening illness perception was more strongly related to DRD in women and younger individuals with T2DM in this cross-sectional study. Though requiring validation, our findings therefore highlight possible targets for risk stratification.

## 1 Introduction

Diabetes mellitus is a chronic metabolic disease with a rapidly increasing global prevalence, expected to reach 1.3 billion by 2050, of which the vast majority (96%) concerns type 2 diabetes mellitus (T2DM) (1). Management of T2DM is achieved through a combination of glucose-lowering medication and lifestyle intervention. Due to its daily management and anxiety over complications among others, T2DM can lead to considerable emotional distress and concern, also known as diabetes-related distress (DRD) (2). DRD encompasses, among other factors, concerns or anxiety about the treatment regimen, complications, and interpersonal relationships (3). DRD prevalence in people with T2DM varies widely (20 to 36%) (3, 4) Psychological health– and thus DRD – plays a key role in diabetes management: DRD may interfere with problem-solving and medication adherence among others (5). As such DRD has been linked to poorer self-care, worse glycemic control, and an increased risk of complications (6–8), making understanding and tackling underlying factors crucial.

Although DRD risk is affected by many different factors, such as a lack of social support or presence of complications (9, 10), an important, modifiable one are the beliefs that individuals hold about the disease itself – otherwise known as illness perceptions. According to the Common-Sense Model of Self-Regulation these consist of beliefs about the cause, timeline, consequences and curability/controllability of an illness (11), and thereby directly shape how individuals cope with their disease (12). Negative or threatening illness perceptions have been linked to both the presence and the severity of DRD in T2DM (13–16), with perceived control being one of the most important dimensions (14, 17). However, most studies on illness perceptions and DRD have been performed in small samples (less than 500 people) (13, 14, 16, 18), and could therefore not assess possible effect modification by sociodemographic factors. Furthermore, perceived causes of T2DM, such as genetics or lifestyle, likely affect the concern and distress individuals attribute to the condition. However, most studies have relied on ‘prompted’ rather than open-ended questions (e.g. rating how much genetics has contributed) (13), which may bias results, or have been conducted on a small scale (n>30) (19).

Although (some aspects of) illness perception and DRD might overlap, particularly regarding concerns and consequences of the disease, the constructs themselves are theoretically different – i.e. illness perceptions refer to how T2DM is perceived, rather than its day-to-day burden (3, 20, 21). While they are theoretically distinct, the constructs are not completely independent. According to the Common-Sense Model, maladaptive illness perceptions (e.g. believing diabetes has severe consequences or is uncontrollable) can contribute to greater DRD (22). Thus, illness perceptions are often studied as antecedents or predictors of DRD rather than being considered the same construct.

Previous studies have reported age, sex and socioeconomic position (SEP) as important (independent) determinants of both DRD and illness perceptions in T2DM, but lack details on how they influence the relation between them. Overall, women seem to have higher levels of DRD (23, 24) and perceive more negative consequences of T2DM, while men have more confidence in treatment by healthcare professionals (25). Furthermore, younger age has been related to more DRD (26), and it is commonly believed that age influences illness perceptions – for example through improved knowledge and awareness with advancing age. Lastly, SEP influences both DRD and illness perceptions with studies showing that 72.5% of people with a primary educational level had DRD compared to 66.7% and 46.4% of those with either a secondary or tertiary level, respectively (27). Regarding illness perceptions, people with a low income or education compared to a high one reported perceiving substantially more negative consequences of T2DM, had less confidence in controlling their T2DM, and had less faith in their medical treatment, among others (28). In addition to their independent associations with illness perceptions and DRD, sociodemographic characteristics may also influence how individuals interpret and respond to illness-related beliefs. For example, differences in coping styles, life circumstances, and available resources across gender, age, and socioeconomic groups may influence the extent to which negative illness perceptions translate into emotional distress and thus modify the strength of the association.

Not only are both DRD and illness perceptions affected by sociodemographic factors, but T2DM risk as well as its management themselves are significantly influenced by age (29), gender/sex (30), and SEP (31, 32) as well, making effect modification likely. Improved understanding of these associations and modifiers could aid in risk stratification and allow for better – more targeted – interventions to decrease DRD and improve the quality of life of people with T2DM in the future. Nonetheless, formal interaction analyses remain largely unreported due to limited sample sizes. Therefore, this study aims to investigate not only the relationship between illness perceptions and DRD, but also how this differs by age, gender, and SEP. We expect that being younger, being a woman, and having a lower SEP makes the association between negative illness perceptions and DRD stronger. In addition, we will provide insights into how perceived causes of T2DM influence DRD by analyzing self-reported causes of T2DM in a large cross-sectional study. Here, we believe that people who claim more personal responsibility in the development of their disease – e.g. lifestyle-related causes – would have higher DRD compared to those attributing it to non-modifiable factors.

## 2 Subjects, materials and methods

### 2.1 Participants

For the current analysis, we used previously collected data from the cross-sectional, self-reported questionnaire study from the public-private collaboration (“Publiek Private Samenwerking; PPS) Lifestyle dIabetes coGnitive beHavioral Therapy (LIGHT) project of the Tilburg University in the Netherlands. As part of this project, quantitative data were collected from people with T2DM via convenience sampling in order to identify barriers and facilitators of a healthy lifestyle. People were eligible for participation if they were 1) aged 18 years or above, 2) had a self-reported diagnosis of T2DM, and 3) had sufficient command of Dutch or English to provide informed consent. In order to decrease the burden on participants, participation was possible through an online link. In recognition of the possible selection bias if participation is only possible through an online platform, we also offered in-person or telephone-based interviews together with a research assistant for participants. During these sessions, questions were asked in the exact same order and way, but study assistants filled it in for participants to ease the burden. Recruitment occurred through various targeted channels including hospitals, general practitioners (GPs) and patient organizations, as well as more general ones, such as advertisements, social media, religious institutions, food banks, and (social) workplaces. By also including verified cases via GPs and hospitals, we also aimed to decrease any possible bias that might occur by relying on a self-reported T2DM diagnosis. Ethical approval was obtained from the Ethical Research Board of the Tilburg University as well as the Medical Ethical Committee (METC) Brabant (NL87355.028.24) and we adhered to the principles of the Declaration of Helsinki.

**Figure 1:**
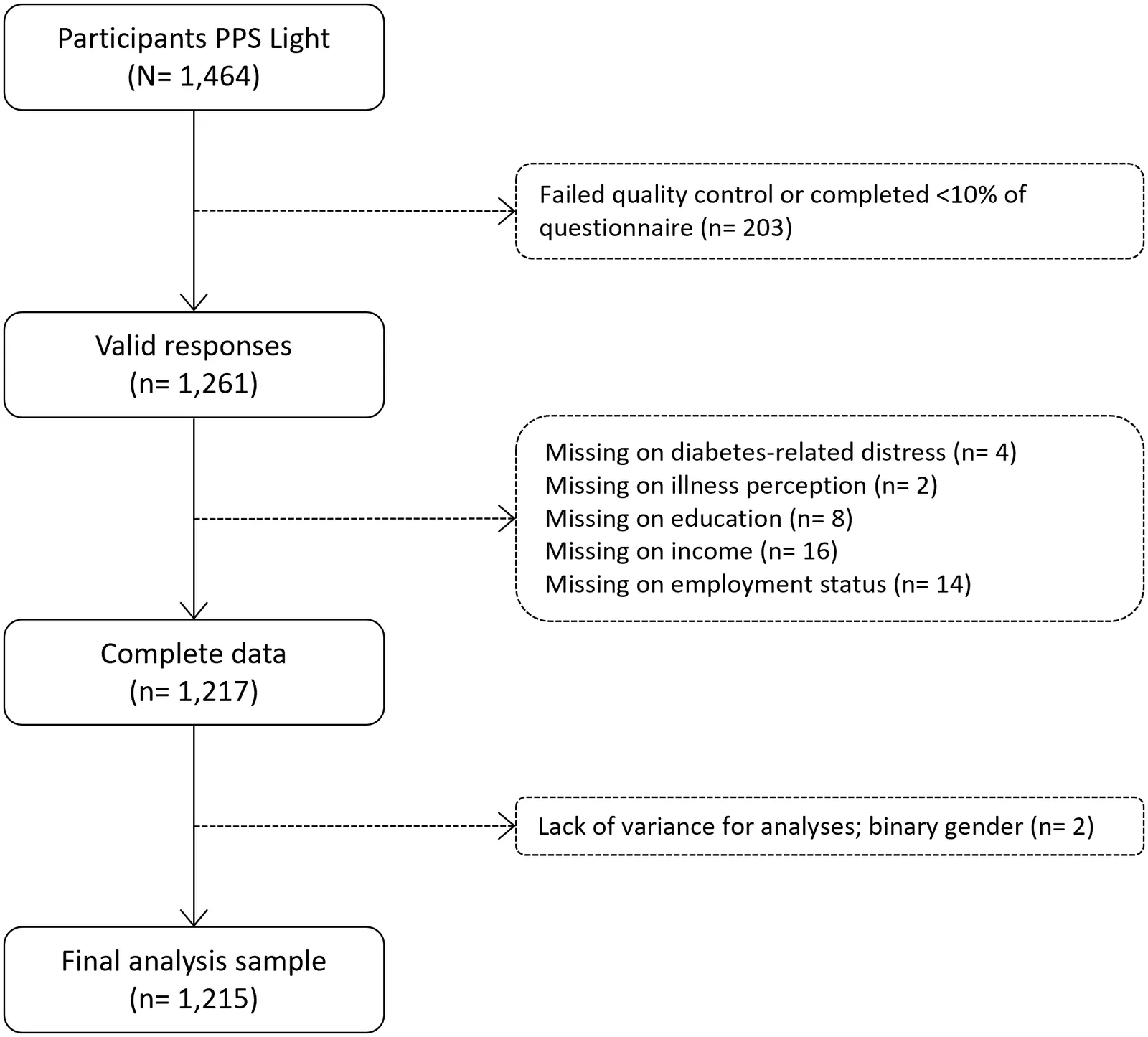
Flowchart showing participant selection.

Of 1,464 participants that provided informed consent between May 2024 and January 2026, 1,261 completed the questionnaire for at least 10% and had valid responses. For the current analysis, we included people with complete data on DRD (n= 1,257), illness perception (n= 1,255), age and gender (n= 1,255), and SEP (1,217). Lastly, two non-binary people were excluded due to the lack of variance within this group for statistical analyses, resulting in a final sample size of 1,215 (**Figure 1**).

### 2.2 Measures

#### 2.2.1 Demographics

Participants were asked to report their gender (woman/man/non-binary/other), age (years), and ethnicity. Ethnicity (Dutch, Other European, Indonesian, Turkish, Moroccan, Surinamese, Caribbean, Chinese, Iraqi, other) was classified into three categories (Dutch, Other European, Non-European). In addition to these general demographics, people were also asked about their age at diagnosis, which was used to calculate T2DM duration (years), the medications they used for T2DM (tablets, insulin, multiple, other, none), whether their HbA1c was controlled (yes/no/don’t know/prefer not to answer), and whether they had ever been diagnosed with the five more common physical (cardiovascular disease (CVD), neuropathy, kidney disease, eye damage, dementia) or psychological (depression, anxiety, eating disorder, bipolar disorder, personality disorder) comorbidities.

#### 2.2.2 Diabetes-related distress

We used the previously developed and validated Problem Areas in Diabetes Scale (PAID)-5 to measure DRD (33). In brief, the PAID-5 includes 5 items regarding different aspects of diabetes-related distress, such as worrying about serious complications and feelings of anxiety about T2DM. Each question is rated on a 5-point Likert scale ranging from 0 (“Not a problem”) to 4 (“A serious problem”). All questions were summed up to create a total score (range 0-20), where a higher score indicates higher levels of DRD. With a Cronbach’s alpha of 0.89 the PAID-5 was considered to have good internal consistency in the current study.

#### 2.2.3 Illness perception and perceived causes of T2DM

The Brief Illness Perception Questionnaire (B-IPQ) was used to measure illness perception (34), which includes nine questions on the impact of and beliefs about people’s illness, such as how long they believe the disease will last, how much control they perceive to have over it, or how concerned they are about it. Q1 concerned consequences of T2DM, Q2 temporality, Q3 personal control, Q4 treatment control, Q5 symptom burden, Q6 concern, Q7 illness coherence, Q8 emotional response, and Q9 the perceived causes of disease. The first eight of these are rated on a 0 (e.g. “No influence”) to 10 (e.g. “A lot of influence”) Likert scale, while the last question is an open-answer item on the three most important perceived causes of a person’s disease. For the purposes of this study, all questions referred specifically to T2DM. A total score was calculated by reverse scoring three items (Q3, Q4, Q7) before summing them up (range: 0-80), with higher scores reflecting a more negative or threatening view of T2DM. The three perceived causes of people’s disease were evaluated and classified into four broad categories, namely ‘Non-modifiable’ (e.g. genes), ‘Lifestyle’ (e.g. obesity), ‘Psychological’ (e.g. stress), and ‘Other’ (e.g. medical malpractice, unknown). For example, if a participant wrote down ‘Father had T2DM’ or ‘Family history’, their cause was classified as ‘Non-modifiable’, whereas answers like ‘Lots of candy’ or ‘Ate too much’ were grouped into the ‘Lifestyle’ category. The B-IPQ has previously been validated in populations with T2DM (35), and was considered to have a good internal consistency in the current study (Cronbach’s alpha: 0.72).

#### 2.2.4 Socioeconomic position

As SEP is hard to define with a single measure, this study included three indicators, namely education, income and employment status. For education, we divided the self-reported highest obtained degree into a low (none/primary school/lower vocational training), middle (secondary general education/secondary vocational training/higher general education), high (higher vocational training/university), or other/non-conventional (don’t know/other education) class. Household income was assessed using a 19-class ordinal scale ranging from <750 to >5,000 euros per month. The income equivalent was calculated by assigning midpoints to each category, 600 to the lowest, and 6,000 to the highest, and subsequently dividing it by the square root of the household size to account for household composition, in line with previous recommendations (36) and similar to the Dutch Maastricht study (37). Income was then divided into three categories based on tertiles, with a separate category for ‘Prefer not to answer’ (n= 216). For employment status, we distinguished between employed (fulltime, parttime), unemployed (unemployed, unfit for work, student, houseman or housewife), and retired.

### 2.3 Statistical analysis

Means and standard deviations (SDs) were calculated for continuous variables, and percentages for categorical variables for the total sample and separately by gender, age, and SEP. T-tests (continuous) and chi-squared tests (categorical) were run to compare all variables across sociodemographic factors. Outliers were checked to assess whether they were within the plausible range. As these were all within the possible range of the questionnaires, they were considered true values rather than measurement error and therefore included in the analysis.

Next, we quantified the relation of the (standardized) total and subdomain scores of illness perception as well as the perceived causes of T2DM (independent variables) with DRD (dependent variable) using linear regression models, adjusting for age, gender, education, income, and employment status in model 1 and additionally for years living with T2DM in model 2. Due to the outcome (DRD) being continuous, and linear regression models being robust in large samples, we used a linear regression model. Illness perception scores were standardized to enable comparison between each sub-domain and the total score. For the illness perception scores, we included interaction terms for all SEP indicators, gender, and age, and ran stratified analyses if significant (p<0.05). Stratification was based on pre-defined groups for gender (women/men), age (<60, 60-69, 70+), education (low/middle/high), and employment (unemployed/pensioned/employed), and on tertiles for income equivalent (euro/month), with an additional category for non-responders (166-1375/1,376-2,526/2,527-6,000/no answer). Due to the low number of people in the ‘Other’ education group, these were removed in the interaction analyses.

As DRD contained a substantial number of ‘0’s (19.5%), and was non-normally distributed even after log-transformation, we not only ran a linear regression, but also included a hurdle model as a sensitivity analysis to test the robustness of our main analysis. As such, we tested whether the total and sub-scores of illness perception were related to 1) the presence of DRD (zero vs non-zero outcome) and 2) the severity of DRD (among people with a non-zero outcome). In running the model, we assumed a negative binomial distribution.

We corrected for multiple testing using the false discovery rate (FDR) correction and considered a corrected p-value <0.05 statistically significant. All analyses were performed in R (version 4.3.2.).

## 3 Results

### 3.1 Descriptive statistics

The sample (n= 1,215) consisted of 53.8% men, with an average age of 66.3 (SD 10.5) years, and diabetes duration of 13.1 (SD 10.1) years (**Table 1**). Participants were predominantly Dutch (92.6%) and retired (53.9%), with either a middle (47.2%) or high (40.6%) educational background and an average monthly income of 2,103 (SD 1,170) euros. They had an average DRD score of 4.5 (SD 4.2) and illness perception of 38.3 (SD: 11.1). When considering perceived causes of T2DM, the majority believed the most important cause of their disease was lifestyle-related (47.6%) or non-modifiable (38.9%) (perceived cause 1). Of note, there was progressively more missing data in perceived causes of T2DM, with only 3.5% missing in cause 1, 10.8% in cause 2, and 30% in cause 3. Missingness in the three perceived causes differed by education, with people in the lowest and ‘other’ education category having significantly higher missingness for cause 1 (7.0%, 9.1%), cause 2 (16.4%, 31.8%), and cause 3 (39.1%, 40.9%), as well as gender and age, with women and the oldest group (>70 years) having more missingness. For cause 3, people refusing to indicate their income also had more missingness, while people who were employed or pensioned had more missingness in cause 1 and cause 2 (**Supplementary Table 1**).

**Table 1:** Sample characteristics.

Characteristic	Level	Total (n= 1,215)
Gender [men, n (%)]		654 (53.8)
Age [mean (SD), years]		66.3 (10.5)
Education [n (%)]	Low	128 (10.5)
	Middle	572 (47.1)
	High	493 (40.6)
	Other	22 (1.8)
Income equivalent [mean (SD)]		2,102.8 (1,168.7)
Employment [n (%)]	Unemployed	182 (15.0)
	Pensioner	655 (53.9)
	Employed	378 (31.1)
Ethnicity [n (%)]	Dutch	1,123 (92.7)
	Other European	21 (1.7)
	Other	68 (5.6)
Years with T2DM [mean (SD)]		13.1 (10.5)
Diabetes medication [n (%)]	Combination	272 (22.4)
	Insulin	85 (7.0)
	Tablets	647 (53.3)
	Other	60 (4.9)
	None	151 (12.4)
HbA1c [n (%)]	Controlled	807 (66.4)
	Uncontrolled	210 (17.3)
	Don’t know	190 (15.6)
	No answer	8 (0.7)
Physical comorbidities [n (%)]	CVD	529 (53.0)
	Neuropathy	298 (29.9)
	Kidney disease	89 (8.9)
	Eye damage	286 (28.7)
	Dementia	9 (0.9)
Psychological comorbidities [n (%)]	Depression	113 (11.3)
	Anxiety	56 (5.6)
	Eating disorder	40 (4.0)
	Bipolar disorder	14 (1.4)
	Personality disorder	19 (1.9)
Diabetes-related distress* [mean (SD)]		4.5 (4.2)
Illness perception** [mean (SD)]	Total score	38.3 (11.1)
	Q1. Consequences	5.1 (2.6)
	Q2. Temporality	9.0 (1.8)
	Q3. Personal control	6.7 (2.0)
	Q4. Treatment control	7.1 (2.1)
	Q5. Symptom burden	4.1 (2.6)
	Q6. Concern	4.7 (2.7)
	Q7. Illness coherence	7.2 (2.1)
	Q8. Emotional response	3.6 (2.7)
Perceived cause 1*** [n (%)]	Non- modifiable	456 (38.9)
	Lifestyle	558 (47.6)
	Psychological	90 (7.7)
	Other	69 (5.9)
Perceived cause 2*** [n (%)]	Non-modifiable	224 (20.7)
	Lifestyle	676 (62.4)
	Psychological	101 (9.3)
	Other	83 (7.7)
Perceived cause 3*** [n (%)]	Non-modifiable	184 (21.6)
	Lifestyle	468 (55.1)
	Psychological	83 (9.8)
	Other	115 (13.5)

*Diabetes-related distress was measured with the PAID-5, where the total score (range 0-20) indicates the amount of diabetes-related distress.

**Illness perception was measured using the B-IPQ, with a possible score between 0 and 80 and higher scores indicating a more threatening view of one’s illness.

***There were 39 missing in cause 1, 121 in cause 2, and 213 in cause 3.

Women, younger people (<60 years), and people who were unemployed or pensioned had a significantly higher DRD and a more negative illness perception compared to men, older people, and those that were employed, respectively (**Supplementary Table 1**). Similarly, people in the highest income group had significantly less DRD and a more positive, non-threatening view of T2DM compared to those who declined to answer or who were in the low- or middle-income group. Participants who were employed were more likely to perceive lifestyle-related factors as the most important cause of T2DM, whereas those who were pensioned or unemployed were more likely to report non-modifiable factors. Regarding age, younger participants reported more psychological or lifestyle-related factors as the most important cause compared to the older groups, who were more likely to report non-modifiable ones. Lastly, while men were most likely to perceive lifestyle as the most important cause (perceived cause 1) of their T2DM (54.0%), women were more likely to attribute it to non-modifiable causes (44.8%) than lifestyle (40.1%).

### 3.2 More negative illness perceptions are related to diabetes-related distress

**Figure 2:**
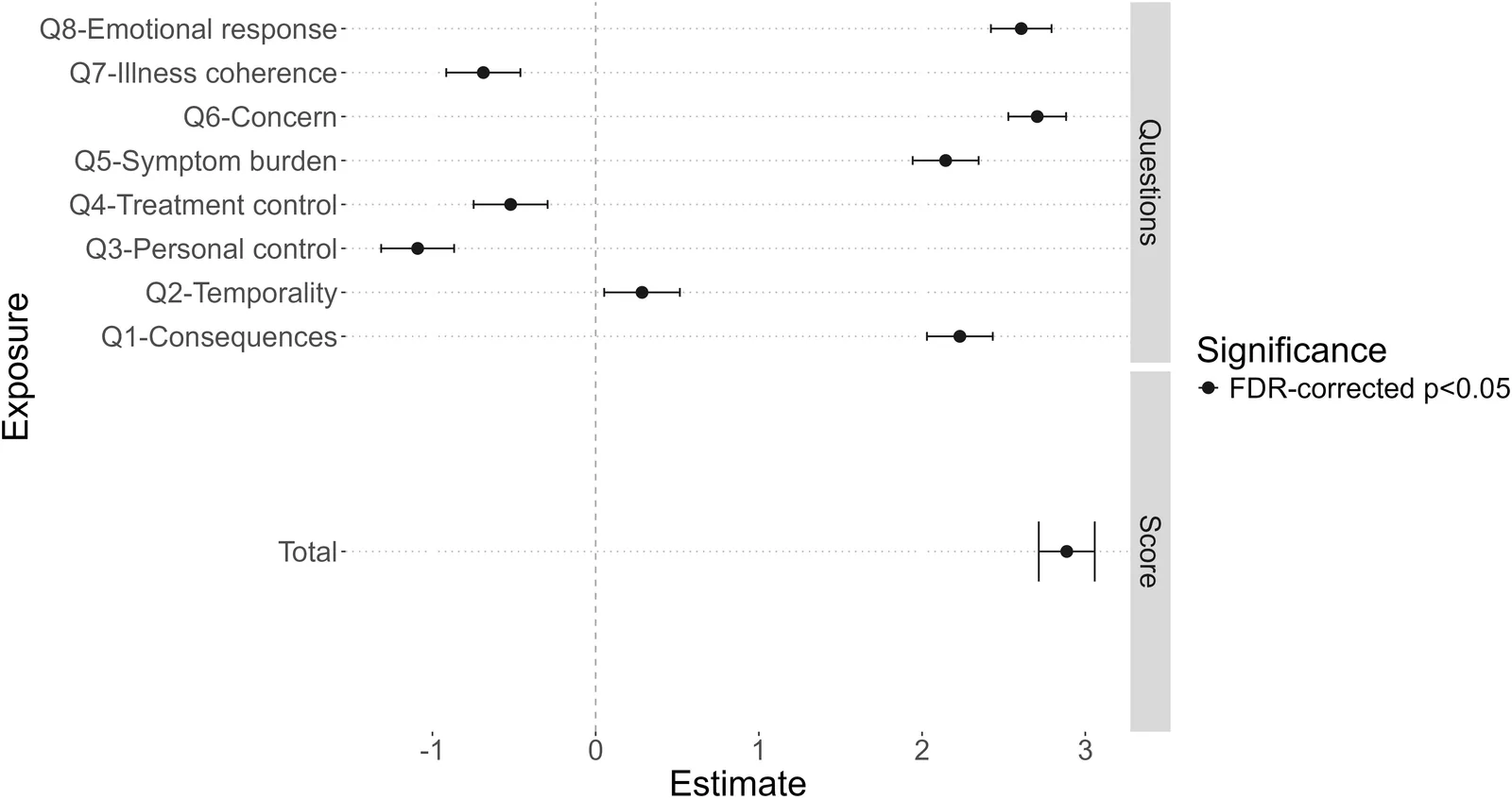
Associations between different aspects of illness perception and diabetes-related distress. Plotted beta estimates (x-axis) for the relation between illness perception (total score, Consequences, Temporality, Perceived personal control, Perceived treatment control, Symptom burden, Concern illness coherence, Emotional response) (y-axis) and diabetes-related distress after adjustment for age, gender, SEP and diabetes duration (model 2). Beta estimates indicate the change in DRD for every SD increase in the respective illness perception score.

One SD increase in the total illness perception score was related to a significant increase in DRD after adjustment for gender, age, SEP, and diabetes duration (Model 2) (**Figure 2**). While all eight sub-questions were significantly associated with DRD, the strongest associations were found between a higher score on Q1 (Consequences), Q5 (Symptom burden), Q6 (Concern), and Q8 (Emotional response) and more DRD. Estimates were similar before and after adjustment for duration of T2DM (**Supplementary Table 2**).

### 3.3 Gender-, age-, and SEP-specific effects of illness perception on diabetes-related distress

**Figure 3:**
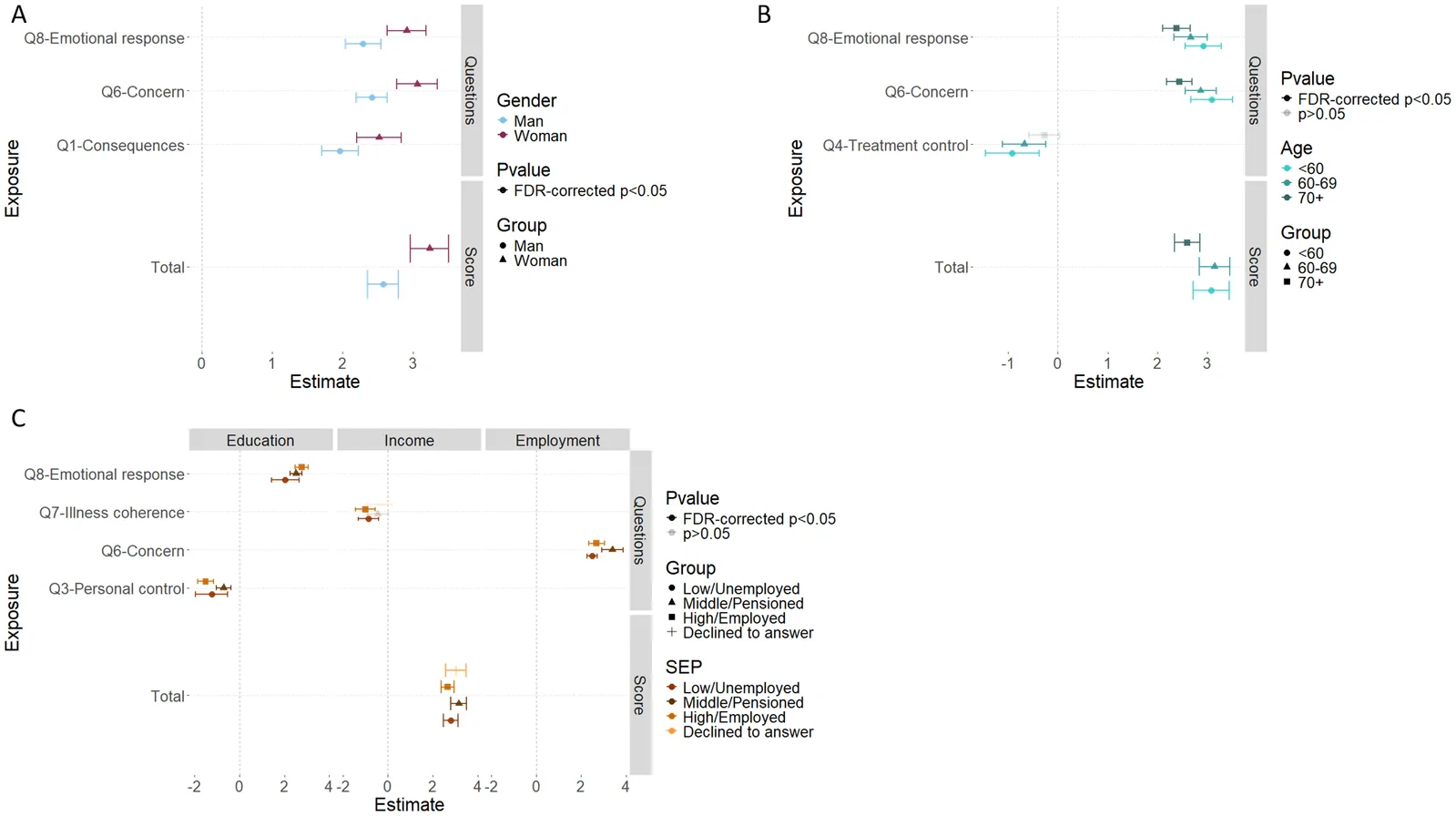
Associations between illness perception and diabetes-related distress by gender, age, and socio-economic position (education, income, and employment status). Where the interaction term was significant for at least one of the group comparisons, stratified analyses for gender **(A)**, age **(B)**, education level **(C)**, income group **(C)**, and employment status **(C)** were run. Stratum-specific beta estimates and 95% confidence intervals (CIs) (x-axis) for the association between illness perception (z-standardized) and diabetes-related distress adjusted for gender, age, education, income, employment status, and diabetes duration are shown. Significance is indicated by transparency.

In the relation between the total illness perception score and DRD, we found that gender, age, and – to a lesser extent – income were significant effect modifiers (**Figure 3**; **Supplementary Table 2**). In model 2, more negative or threatening illness perceptions were more strongly related to DRD in women compared to men, as well as in the two younger (<60, 60–69 years) groups compared to the older one (70+) (**Figure 3**). Regarding income, only the interaction term comparing the ‘Middle’ versus the ‘High’ (reference) was significant, where people in the ‘Middle’ group had a stronger estimate. Associations were similar before and after adjustment for diabetes duration (**Supplementary Table 2**).

Regarding sub-domains of illness perception, results were consistent for gender and age. When comparing women and men, Q1 (Consequences), Q6 (Concern), and Q8 (Emotional response) were more strongly related in women (**Figure 3**). With regards to age, there were significant interactions for Q6 and Q8, as well as Q4 (Treatment control). After stratification, the young age group (<60 years, n= 292) compared to the middle (60–69 years, n= 409) and older (70+ years, n= 514) groups, was consistently shown to experience a stronger association between illness perceptions and DRD after adjustment for gender, SEP and diabetes duration. When considering SEP, there were fewer significant interaction terms: there was significant effect modification for Q3 (Personal Control) in the High/Low education, Q8 (Emotional response) in the High/Middle education, Q7 in the High/No answer income, and Q6 (Concern) for the Employed/Unemployed comparison. Stratified analyses showed largely inconsistent results with associations sometimes being stronger for the ‘Middle’ and sometimes the ‘Low’ or ‘No response’ group. Although most associations were similar before and after adjustment for diabetes duration, the income interaction for Q7 was only significant in Model 2.

An overview of all associations can be found in **Supplementary Table 2**.

### 3.4 Perceived causes of diabetes and diabetes-related distress

When considering the type of causes people attributed to their T2DM, people who believed the most (Cause 1) or second most (Cause 2) important cause of T2DM was psychological in nature compared to a non-modifiable factor such as genetics or age, had significantly more DRD independent of age, gender, SEP, and diabetes duration. Associations were similar before adjustment for diabetes duration (**Supplementary Table 2**).

### 3.5 Sensitivity analysis

Due to the high number of zeros and right-skewed nature of our data, we next assessed the association between the illness perception scores and DRD using a hurdle model, adjusting for gender, age, SEP, and diabetes duration. Comparing people without any DRD (zeros) to people with any DRD (non-zeros), we find similar results to our linear regression. Indeed, one SD increase in the total illness perception score was related to 6.00 times higher odds of having any DRD. Among people with DRD (DRD >0), an increase in illness perception was also related to a higher DRD. While all 8 sub-questions were significantly related to the presence of DRD, Q2 (temporality) was not significantly related to an increase in DRD among people who reported any DRD (non-zeros). Overall, the sensitivity analysis provided similar results to the linear regressions, showing the robustness of our results.

## 4 Discussion

In this large (n=1,215) cross-sectional sample of people with T2DM, we found that a more threatening view of T2DM was related to higher levels of DRD, and that this differed by sociodemographic factors. Across both the total score and subdomains of illness perception, we showed that women, younger people and those that were unemployed or retired had higher mean illness perception scores – i.e. a more threatening view of T2DM – compared to men, older people, or those that were employed. Not only did women and younger people have a more threatening view of T2DM, but we furthermore showed that these perceptions were also more strongly related to DRD. In contrast, while mean levels of both illness perceptions and DRD differed by SEP, there was no consistent evidence of effect modification in the association between the two. Furthermore, in relating perceived causes of T2DM to DRD, we found that people who believed their T2DM was caused by a ‘Psychological’ issue, such as stress, experienced more DRD. Interestingly, missingness increased in a stepwise manner across the three most important perceived causes of disease, where missing answers were the lowest in the first cause (3.5%), higher in the second (10.8%), and highest in the third (30%).

While our results – showing a clear albeit cross-sectional relation between more threatening illness perceptions and DRD –echo previous studies (13–16), we uniquely show interaction effects. Compared to men, women did not only have a more threatening view of T2DM, as shown previously (25), but also seemed more sensitive to it. Indeed, a more negative overall perception, as well as more negative views regarding consequences, concern, and emotional response specifically, were more strongly related to DRD in women. Overall, women tend to suffer from mental illnesses more than men (38, 39), especially when it concerns internalized disorders, such as depression and anxiety (40). In part, this could be due to the higher prevalence of rumination or worrying about the future as a (detrimental) coping mechanism in women (41). If women are more susceptible to these, this may explain not only why they would perceive T2DM as more threatening, but also why more negative perceptions of its consequences, greater concerns about the disease, and its emotional burden are more strongly related to DRD. These stronger associations suggest that risk stratification may be beneficial and highlight illness perceptions as a potential target for future research in decreasing DRD in women.

Furthermore, our results highlight that younger people (<60 years) not only viewed their disease as more threatening, but that this was also more strongly related to DRD. Importantly, all associations were independent of T2DM duration, suggesting that *age* rather than *disease experience* might be crucial. While younger people have previously been reported to have more negative views of T2DM (26, 42), and longitudinal research is necessary for confirmation, our results could suggest they might be more vulnerable as well. We speculate that an explanation for this might lie in the social environment. Due to the strong age-component in T2DM risk (29), older populations might be more likely to know others with T2DM or similar conditions, whereas younger people might lack these peers. Peer support has been shown to be effective in reducing DRD and improving T2DM management (43), and the social environment directly impacts someone’s beliefs about their illness (12). Lacking peers could therefore negatively impact both DRD and illness perceptions. Although there is no evidence yet to support this, we think it is possible that in comparing themselves to their (healthier, mostly disease-free) environment, younger people with T2DM might consider themselves unhealthier, and come to view T2DM as well as its risks in a more threatening manner. Without receiving personal support, advice, or knowledge from peers, they might furthermore be unable to alleviate concerns as easily compared to older groups, which may in turn lead to increased (diabetes-related) distress. Furthermore, T2DM being generally considered an ‘old-person disease’ might also increase these concerns. More in-depth research on the reasons behind these age differences is necessary to confirm these hypotheses.

Unexpectedly, we found no substantial SEP-specific effects of illness perceptions on DRD. Although we confirmed that people with a low SEP generally had a more threatening view of T2DM (28), particularly when using employment status as a proxy of SEP, there was little evidence of effect modification across SEP groups. Overall, our findings on SEP thus suggest that people with a lower SEP might be at increased risk of DRD through more negative illness perceptions, but that the strength of the association does not differ between groups.

In addition, we assessed open-ended questions on perceived causes of T2DM, and found that people attributing T2DM to psychological factors had higher levels of DRD. This could be due to a shared cause of both: people who already suffer from mental distress, might be more likely to develop diabetes-related distress as well. Both T2DM and mental disorders or psychopathologies share several risk factors, such as severe childhood events and rumination (44–46). Psychological distress may also translate to diabetes-specific concerns: a spillover effect. After all, if people already suffer from psychological distress before they get diagnosed with T2DM, they might also have little cognitive load to cope with T2DM, and threatening perceptions might therewith have a stronger effect on DRD. Notably, there was consecutively more missingness in reporting the three most important causes of T2DM. Despite T2DM pathogenesis being complex, and there being a multitude of risk factors (47), patients themselves seem to be less likely to attribute more than one or two causes to their disease. Although an interaction analysis investigating effect moderation by age, gender, and SEP was not performed for the association between perceived causes of T2DM and DRD, we did observe that successive missingness differed by these factors. Indeed, older people and people with a lower SEP tended to have more missing in the perceived causes of T2DM.

Our study has several key strengths. Firstly, this study combined a large sample size (with an almost equal number of women and men) with a comprehensive analytic approach, allowing us to examine both dimensional illness perception scores and categorical attributions of perceived disease causes, which are often overlooked. Furthermore, most studies were performed on relatively small samples of ~500 people (13, 14, 16, 18), or provided a wide variety of different determinants of DRD and therefore lacked details on illness perception (15). By assessing more than 1,200 individuals with T2DM in this cross-sectional study, we not only provided an in-depth analysis of how different dimensions of illness perceptions relate to DRD, but we could also evaluate effect modification by sociodemographic factors, which has resulted in novel insights in the association between illness perception and DRD. As such, these might highlight potential individuals at risk, and targets for future research.

Nonetheless, several limitations should also be considered. In classifying and coding perceived causes of T2DM, it was clear that some people struggled with understanding the question. For example, people wrote down symptoms of T2DM (thirst) or its diagnostic criterium (hyperglycemia). This might especially affect generalizability of our results to people with a lower SEP, as people with a lower (health) literacy are more likely to misread or misunderstand the question. As such, their responses are more likely to be missing. People with a lower education, which is somewhat related to health literacy, were indeed found to have higher missingness in these questions – suggesting they either did not understand the question, provided more incorrect responses, or did not perceive any other important causes of their disease. Secondly, there was a lack of ethnic diversity in our population, with over 90% of the participants being Dutch, which limits generalizability to non-Western populations. Not only is T2DM prevalence higher among ethnic minorities (48), but they also experience higher levels of DRD (49) and view and manage T2DM differently (50). For example, a previous study showed that Javanese people in Indonesia had some misconceptions around T2DM (e.g. the existence of a more moderate ‘dry’ versus a more dangerous ‘wet’ sugar type), held different beliefs about its treatment (e.g. use of medicinal plants), and had different coping mechanisms (e.g. managing stress by submitting to God) (51). Different cultural beliefs might clash with the dominant healthcare system itself, which makes differential effects of illness perception (e.g. belief in treatment) on DRD by ethnicity likely. However, our sample was not diverse enough to investigate these differences, nor should results be considered generalizable to these groups. Thirdly, T2DM diagnosis was self-reported, which might have led to the inclusion of people without a verified diagnosis. While part of our recruitment occurred through hospitals and GPs, and we consider it unlikely that people misreport their official diagnosis based on previously reported concordance between a self-reported T2DM diagnosis and medical records (52), it is certainly possible that a couple of people in our sample do not actually have T2DM. Insofar as this would have influenced our results, we believe the effect will be minimal due to the large overall sample size and the proportion of verified diagnoses. Certain participant characteristics, such as the age at diagnosis, also provided additional confirmation that it is T2DM rather than, for example, Type 1 Diabetes Mellitus (T1DM). Fourthly, open-ended responses regarding perceived causes of T2DM exhibited cumulative missingness reaching 30% among older participants and those with lower SEP. Consequently, descriptive frequencies of illness attributions in these specific sub-groups must be interpreted with caution, as they are subject to potential non-response bias. Lastly, both recall/reporting bias and selection bias might have influenced our findings. After all, social desirability might have made people report a more positive view of T2DM and less DRD, and self-selection might have led to a lack of people who are struggling the most with their views on T2DM: although our results show no difference in adjustment for diabetes duration, people might have been less likely to participate in the study if they have not yet accepted the diagnosis. Insofar as this would have impacted our results, we expect the association to be stronger in the actual population, where variance is larger. Similarly, selection bias – where people with a lower SEP are less likely to participate – might have weakened our power to detect significant SEP interactions. Although we did find that people with a lower SEP had significantly more DRD and a more threatening view of T2DM, we might have therefore lacked the power to find a difference in the strength of the association.

## 5 Conclusions

In conclusion, we leveraged data from a large cross-sectional study to answer the question of whether a more threatening illness perception was related to DRD and how this relationship differed by age, gender and SEP categories. Women, younger people (<60 years), and people with a lower SEP have higher levels of DRD compared to men, older people and high SEP, but we uniquely show that age and gender, but not SEP, consistently and significantly modified these associations. Specifically, these associations might be stronger in younger people and women. Secondly, we aimed to assess how perceived causes of T2DM were related to DRD. With previous studies limited in either sample size, or a pre-defined set of questions (rather than a less biased, open-answer prompt), we provided novel insights into the perceptions around T2DM as well as its relation with DRD. Indeed, we observed that people usually attributed their disease to only one or two factors, despite the multifactorial nature of T2DM pathogenesis. In relating perceived causes of T2DM to DRD, we found that attributing T2DM to psychological causes was related to higher levels of DRD. As such, addressing these beliefs could be a potential avenue for reducing DRD.

Although further validation of our findings is necessary in a more clinical and longitudinal setting, as well as a culturally diverse sample to improve generalizability to populations of a non-Western origin, our results highlight potential avenues for risk stratification and the importance of targeted interventions in women and younger adults to reduce DRD.

## Data Availability

The data that support the findings of this study are not openly available due to privacy reasons and are available from the corresponding author upon reasonable request. Data are located in controlled access data storage at Tilburg University. Requests to access the datasets should be directed to S.S.Soedamah@tilburguniversity.edu.
